# SSTR Antagonists as Theranostic Option in Merkel Cell Carcinoma

**DOI:** 10.2967/jnumed.123.267124

**Published:** 2024-06

**Authors:** Malte Kircher, Adriana Amerein, Mareike Augustin, Nic G. Reitsam, Johanna S. Enke, Marianne Patt, Georgine Wienand, Ralph A. Bundschuh, Christian H. Pfob, Constantin Lapa, Alexander Dierks

**Affiliations:** 1 Department of Nuclear Medicine, Faculty of Medicine, University of Augsburg, Augsburg, Germany;; 2 Department of Dermatology, Faculty of Medicine, University of Augsburg, Augsburg, Germany; and; 3 Department of Pathology, Faculty of Medicine, University of Augsburg, Augsburg, Germany

Merkel cell carcinoma is a rare, highly aggressive skin cancer. With multimodal treatment including chemo- and immunotherapy, the 5-y overall survival ranges from 14% to 62%, depending on the disease stage at diagnosis (1). New treatment options are therefore urgently needed. Given the overexpression of somatostatin receptors (SSTRs) due to its neuroendocrine features, SSTR-directed therapy could be a promising target in metastatic Merkel cell carcinoma (2–4).

To further investigate this potential, 2 clinical trials are already ongoing in which peptide receptor radionuclide therapy with SSTR agonists are being studied in combination with immunotherapy (GoTHAM trial, NCT04261855; iPRRT trial, NCT05583708).

Although various agonistic SSTR-targeting tracers have been established for years in metastatic Merkel cell carcinoma and other neuroendocrine tumor entities, tracers with antagonistic receptor interaction are recognized as a new, promising theranostic option, as they can achieve high tumor uptake and prolonged retention as compared with agonists (5).

We report the case of a 77-y-old man with recurrent metastatic Merkel cell carcinoma who underwent PET/CT with the ^68^Ga-labeled SSTR antagonist SSO120 (international nonproprietary name: satoreotide trizoxetan; also known as NODAGA-JR11, OPS202, and IPN01070; injected dose, 160 MBq; scan acquisition, 60 min after injection) (6*,*^7^) to explore the possibility for peptide receptor radionuclide therapy (Fig. 1A). Informed consent was obtained from the patient. Compared with [^18^F]FDG PET (Fig. 1B), a more intense tracer uptake and excellent tumor-to-background ratios were observed using [^68^Ga]Ga-SSO120 PET, for example, in a pelvic (right iliac) lymph node metastasis with an SUV_max_ of 11.6 versus 5.5 on [^18^F]FDG PET. The average SUV_max_ in the 6 measurable tumor lesions was 13.4 ± 5.0 with [^68^Ga]Ga-SSO120 versus 9.5 ± 4.2 with [^18^F]FDG PET. Given the still-localized tumor stage, the patient underwent surgery. High membranous SSTR expression on all tumor cells was confirmed by immunohistochemistry (score 3+; Fig. 1C).

**FIGURE 1.**
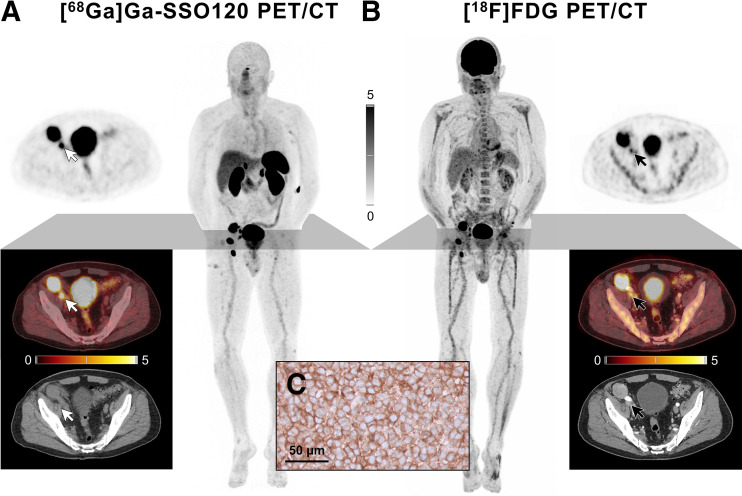
Maximum-intensity projections and axial sections of [^68^Ga]Ga-SSO120 (A) and [^18^F]FDG (B) PET/CT. Location of exemplary pelvic (right iliac) lymph node metastasis with SUV_max_ of 11.6 vs. 5.5 on [^18^F]FDG PET is indicated by white and black arrows, respectively. Intensity scale bars are SUV. Immunohistochemistry showed high membranous SSTR expression on all tumor cells (score 3+; C).

In conclusion, PET/CT with SSTR antagonists could serve as a noninvasive read-out for tumor biology and allow selection of candidates for SSTR-directed peptide receptor radionuclide therapy. Further research, especially regarding advantages over agonistic vectors, is highly warranted.

## DISCLOSURE

No potential conflict of interest relevant to this article was reported.
